# Relationship between choroidal thickness changes and macrophage polarization in high myopia

**DOI:** 10.3389/fmed.2026.1826072

**Published:** 2026-04-22

**Authors:** Zhou Zhou, Wen Long, Bingru Zheng, Dongmei Cui

**Affiliations:** 1 The Second Clinical Medical College of Jinan University, Shenzhen Eye Hospital, Shenzhen, China; 2 Shenzhen Eye Hospital, Shenzhen Eye Medical Center, Southern Medical University, Shenzhen, China

**Keywords:** choroidal thinning, high myopia, immune-inflammatory mechanisms, macrophage polarization, macrophages

## Abstract

High myopia is one of the major causes of blindness worldwide, and the pathological mechanism underlying its characteristic progressive choroidal thinning has long been a research focus. Traditional views have mostly attributed choroidal thinning to the passive result of mechanical stretch caused by axial elongation or insufficient choroidal blood perfusion. With deeper research, increasing evidence suggests that an active and dynamic immune-inflammatory remodeling process may exist in the choroid. By systematically reviewing recent findings in myopia and immunology, and integrating evidence from clinical imaging, intraocular fluid analyses, and animal experiments, this review attempts to construct a pathological framework centered on the following core sequence: “mechanical stretch/relative hypoxic stress–immune microenvironment imbalance–macrophage polarization–destructive choroidal remodeling.” This article focuses on how macrophages may become polarized after sensing mechanical stretch and hypoxic signals, and how these changes may contribute to choroidal structural and functional degeneration through cytokine secretion, regulation of matrix metabolism, and mediation of vascular regression. In addition, this review summarizes areas that still need improvement, including human tissue data and evidence from macrophage subset-specific interventions, and discusses future directions such as spatial transcriptomics and targeted immune modulation, with the aim of deepening the understanding of the pathological mechanisms of high myopia and providing references for the development of new prevention and treatment strategies.

## 1 Introduction

### 1.1 Epidemiology and disease burden of high myopia

Myopia is a major public health problem worldwide in the 21st century. The latest epidemiological data show that approximately 30%–50% of the global population suffers from myopia, and in some East Asian countries or regions, the prevalence of myopia in adolescents is even higher than 80%. The prevalence of high myopia (HM) is also showing a significant upward trend. It is estimated that by 2050, the number of people with myopia worldwide will reach about 5 billion, and the number of people with high myopia will reach 1 billion. Myopia-related visual impairment may further increase, becoming one of the most common irreversible causes of blindness (1).

High myopia is usually defined as a refractive error greater than −6.00 D or an axial length of 26.00 mm or more. According to the definition of the International Myopia Institute (IMI), pathologic myopia (PM) is a special type of myopia characterized by excessive axial elongation accompanied by progressive thinning of the posterior choroid. This leads to degenerative structural changes in the posterior segment tissues–including the retina, choroid, and sclera– ultimately causing irreversible impairment of best-corrected visual acuity (BCVA) (2), seriously threatening visual health and quality of life. Statistics indicate that approximately 30%–70% of high myopia individuals will progress to pathologic myopia (3). HM is typically defined in terms of refractive status or axial length, whereas PM places greater emphasis on structural lesions of the posterior segment resulting from excessive axial length. It should be noted that HM and PM are not entirely equivalent: HM is primarily a refractive/biometric concept, whereas PM is a spectrum of structural diseases characterized by typical retinal pathologies. Therefore, in the following text, the term HM is primarily used when referring to simple refractive/axial length stratification, while PM is primarily used when referring to structural complications.

The choroid provides the main oxygen and nutrient supply to the outer retina and is essential for maintaining normal visual function. Choroidal thinning is a characteristic structural change in pathologic myopia and is closely associated with degenerative alterations of posterior segment tissues. It is considered an important pathological factor contributing to visual impairment (4).

Early animal experiments demonstrated that the choroid has rapid and reversible dynamic regulatory capacity. For example, in chick models, the choroid thickens when the eye is under myopic defocus, whereas hyperopic defocus causes thinning (5, 6). Similar findings were subsequently reported in guinea pig, marmoset, mouse, and tree shrew models. Collectively, these observations indicate that the choroid is not merely a structural component of the globe, but an active participant in myopia development. Its mechanisms deserve in-depth study, and these data also provide an important foundation for subsequent cellular and molecular investigations of its regulatory mechanisms.

With the widespread application of enhanced depth imaging optical coherence tomography (EDI-OCT) and swept-source optical coherence tomography (SS-OCT), *in vivo* choroidal structure can now be quantitatively evaluated at high resolution, which has greatly promoted progress in myopia-related choroid research. Numerous clinical studies have suggested that choroidal thinning is a common pathological phenomenon in myopic patients and is significantly associated with axial elongation and increased refractive error (7, 8). In particular, in highly myopic eyes with posterior scleral staphyloma, the choroidal thickness in the macular and posterior pole regions is significantly thinner than that in emmetropic eyes or eyes with low-to-moderate myopia, and the degree of thinning is negatively correlated with axial length (9–11). Consistent with this pattern, thinner choroid in the macular and posterior pole regions has also been reported in real-world highly myopic cohorts (12). Early classical studies using spectral-domain OCT found that in highly myopic eyes with axial length > 26.0 mm, subfoveal choroidal thickness was approximately half that of normal eyes, with concordant thinning across multiple posterior pole locations (4). Overall, the longer the axial length, the thinner the choroid (13–15); each 1 mm increase in axial length is associated with an approximately 20–60 μm reduction in choroidal thickness (16). Moreover, choroidal “thinning” is not limited to thickness reduction, but is also accompanied by a decrease in vascular components. A cross-sectional study using EDI-OCT combined with modified Niblack image binarization divided the choroid into a “black” luminal area (representing vessels) and a “white” stromal area (representing connective tissue and others), and suggested that the reduction in choroidal thickness with axial elongation was mainly associated with a relative decrease in luminal area (17). Recent studies further show that the choroidal vascularity index (CVI) is significantly lower in highly myopic eyes than in emmetropic eyes, and that progression of myopia is accompanied by changes in the spatial distribution and tissue volume of choroidal vessels, supporting a trend toward structural degeneration (18). Therefore, OCT-based choroidal thickness and vascular-related parameters provide important quantitative markers for evaluating myopia progression and choroidal status.

Therefore, OCT-based choroidal thickness and vascular-related parameters provide important quantitative markers for evaluating myopia progression and choroidal status. From a translational perspective, the proposed choroid-focused remodeling model should be interpreted in relation to commonly used clinical imaging metrics. Choroidal thickness remains the most widely used structural parameter, but both subfoveal and regional measurements should be considered, because highly myopic eyes–particularly those with PM/myopic macular degeneration (MMD) or posterior staphyloma–often show substantial topographic heterogeneity rather than uniform thinning. In this context, the choroidal vascularity index (CVI) may provide complementary information by reflecting the relative balance between luminal and stromal components, and may therefore be more closely aligned with vascular rarefaction and stromal remodeling than thickness alone. In parallel, OCTA-derived choriocapillaris/perfusion measures offer a functional dimension that complements structural OCT readouts. OCTA-based studies in highly myopic eyes have shown that microvascular readouts are dynamically altered in clinical settings and may provide complementary functional information beyond structural thickness measurements. For example, postoperative OCTA assessment after ICL implantation demonstrated changes in foveal avascular zone size and regional retinal/peripapillary flow density in highly myopic eyes, further support the broader value of perfusion-oriented imaging metrics as clinically accessible surrogate endpoints in high myopia (19). Accordingly, if macrophage-associated inflammatory remodeling contributes to myopic choroidal degeneration, its clinical correlates would more plausibly be expressed as a coupled pattern of regional or subfoveal choroidal thinning, altered CVI, and impaired choriocapillaris/perfusion signals, rather than as an isolated change in a single imaging metric. At present, however, these imaging parameters should be regarded as translationally relevant surrogate endpoints rather than direct readouts of macrophage state.

### 1.2 Discussion of traditional theories of myopia pathogenesis

The clinical manifestations of high myopia and related choroidal thinning have been well characterized, but the underlying cellular and molecular mechanisms remain incompletely understood, especially the key question of why the choroid shows active and progressive thinning and remodeling, which remains a major focus and challenge in current research.

**FIGURE 1:**
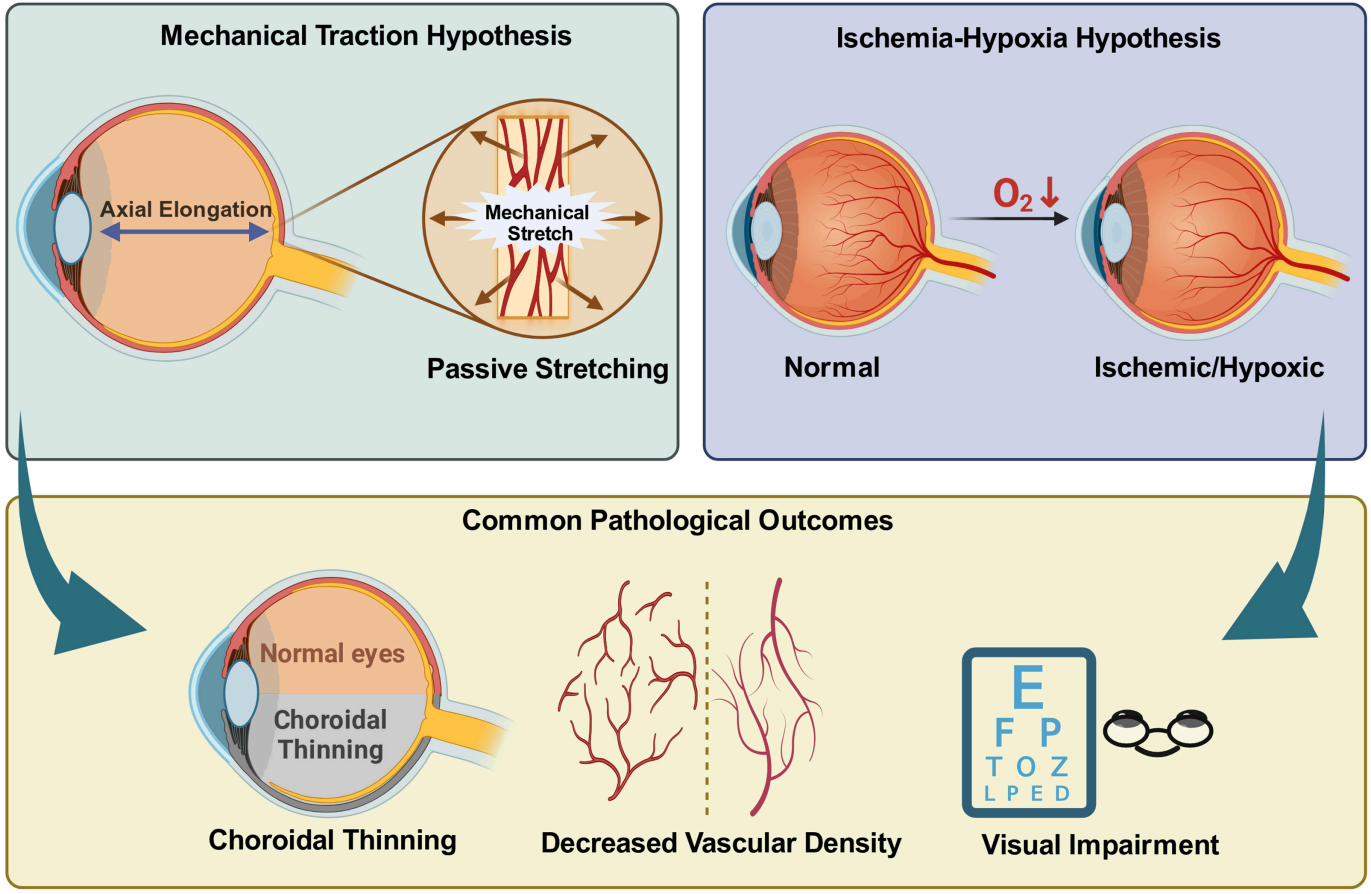
Traditional hypotheses of choroidal thinning in high myopia. The mechanical traction hypothesis attributes choroidal thinning to passive stretching caused by axial elongation, whereas the ischemia-hypoxia hypothesis emphasizes reduced choroidal perfusion and relative hypoxia. Despite different upstream mechanisms, both hypotheses converge on common pathologic outcomes, including choroidal thinning, reduced vascular density, and visual impairment. Created with BioRender.com.

In early studies of myopia pathogenesis, the “mechanical stretch hypothesis” and the “ischemia-hypoxia hypothesis” were dominant. They provided important frameworks for understanding axial elongation and tissue structural changes. However, with deeper research, the active and progressive thinning of the choroid in HM and the associated complex biological responses still require further discussion Figure 1.

#### 1.2.1 Discussion of the “mechanical traction hypothesis”

This hypothesis emphasizes the physical mechanical stretch imposed on the choroid by pathological axial elongation and attributes choroidal thinning mainly to passive extension. However, a purely mechanical perspective cannot fully explain why choroidal thickness can show predictable dynamic changes at very early stages of myopia and is often accompanied by immune cell recruitment, activation of inflammatory signaling pathways, and systematic changes in matrix remodeling-related factors. The IMI consensus statements also indicate that bidirectional changes in choroidal thickness may precede changes in ocular growth and may predict the direction of axial elongation. These observations support the view that the choroid is not merely a passive structure and suggest that it may have active regulatory functions (7).

#### 1.2.2 Discussion of the “ischemia-hypoxia hypothesis”

This hypothesis emphasizes that axial elongation reduces choroidal blood perfusion, induces tissue hypoxia, and then affects choroidal thickness and metabolism (20). The hypothesis reveals the correlation between choroidal thinning and reduced blood flow, but additional theoretical frameworks are still needed to explain all pathological phenomena observed in highly myopic eyes. Emerging evidence further suggests that the choroidal microenvironment in high myopia is not characterized by a simple “ischemic/hypoxic” state, but rather by a complex pathophysiological process involving “chronic inflammation” (21). Therefore, hypoxia may be an important upstream driver, but its downstream effects likely need to be interpreted together with immune-inflammatory responses, extracellular matrix remodeling, and intercellular communication networks. Integrating these processes may provide a more complete explanation for progressive choroidal degeneration and thinning.

### 1.3 Immune mechanisms involving macrophages

At present, direct immunophenotypic evidence from the human myopic choroid remains limited. Therefore, while this section integrates studies related to the choroid and the RPE–choroid unit, it also incorporates relevant evidence derived from the sclera, retina, and broader immunologic research in order to formulate choroid-focused mechanistic hypotheses. Where the cited evidence is not choroid-specific, it should be interpreted as inferential and hypothesis-generating rather than as direct confirmation of mechanisms in the human myopic choroid.

In recent years, an increasing number of basic and translational studies have suggested that inflammatory and immune mechanisms are not merely accompanying phenomena in the development and progression of myopia, but may also participate in its pathologic progression. As important resident or recruited immune cells within posterior segment tissues, including the choroid, retina, and sclera, macrophages are highly plastic and sensitive to microenvironmental changes. Through cytokine secretion and modulation of local immune status, macrophages may participate in the dynamic remodeling of the posterior segment microenvironment and thereby influence the maintenance or disruption of tissue homeostasis. Accordingly, macrophages may represent an important hub linking abnormal biomechanical stress, metabolic stress, and tissue remodeling (22).

Traditionally, macrophage functional states are often summarized by the “classically activated (M1)–alternatively activated (M2)” model: M1 macrophages are generally associated with proinflammatory responses and tissue injury, whereas M2 macrophages are more commonly linked to anti-inflammatory regulation, tissue repair, and fibrotic remodeling (23, 24). It should be noted that although the M1/M2 dichotomy is useful for discussion, macrophages in real pathological environments often exist along a continuum and in multiple coexisting states, and their functional states are shaped by mechanical stimulation, hypoxia, metabolic substrates, damage-associated molecular patterns (DAMPs), and various cytokines. Current evidence remains insufficient to reliably distinguish tissue-resident macrophages from monocyte-derived macrophages, and it is also difficult to resolve more refined subsets defined by markers such as CCR2, CX3CR1, CD68, CD163, and MHC-II. Therefore, in myopia research, the key question may not be limited to whether macrophages conform strictly to an “M1” or “M2” state, but rather how their state composition, spatial distribution, and effector networks change across different stages of HM and PM.

Evidence for inflammatory mechanisms and macrophage involvement in myopia progression has been supported in multiple animal models. Zheng et al. observed an increased density of M2 macrophages in the sclera of form-deprivation myopic mice, suggesting that modulation of macrophage polarization may influence myopia progression (25). In addition, single-cell transcriptomic study by Yao et al. in a high-myopia-related model showed that resident retinal macrophages/microglia exhibited upregulated inflammatory mediators and TGF-β receptor expression, suggesting a more activated state with increased sensitivity to inflammatory and growth-regulatory signals (26).

Studies of intraocular fluids have demonstrated altered inflammatory mediator profiles in the aqueous humor and vitreous fluid of patients with high myopia. Mérida et al. analyzed aqueous humor samples from highly myopic patients with cataract and found significantly increased levels of multiple inflammatory mediators, including platelet-derived growth factor-BB (PDGF-BB), hepatocyte growth factor (HGF), and interleukin-6 (IL-6), which were positively correlated with axial length and the severity of macular degeneration (27). Similarly, Han et al. reported significantly elevated levels of IL-6, monocyte chemoattractant protein-1 (MCP-1), and vascular cell adhesion molecule-1 (VCAM-1) in the aqueous humor of patients with pathologic myopia or simple high myopia with cataract, compared with nonmyopic controls (28).

Zhang et al. further compared aqueous and vitreous cytokine-related parameters between patients with simple high myopia and those with high myopia complicated by myopic choroidal neovascularization (mCNV). Inflammatory cytokines, including IL-6, IL-10, and MCP-1 (CCL2), were significantly increased relative to nonmyopic controls, with more pronounced elevations in eyes with mCNV. These findings indicate that intraocular inflammatory cytokine upregulation is present in highly myopic eyes regardless of mCNV status, suggesting that a persistent inflammatory microenvironment may be established before overt mCNV develops and may contribute to a permissive microenvironment for VEGF upregulation and subsequent mCNV formation.

Importantly, this study further proposed that cytokine total mass may better reflect pathologic changes in highly myopic eyes than aqueous concentration alone. By integrating aqueous cytokine concentration, axial length, and vitreous volume, the authors estimated the total cytokine mass within the vitreous cavity (aqueous concentration × vitreous volume). Using this approach, they showed that although aqueous VEGF concentrations did not differ significantly between groups, total intraocular VEGF secretion was increased in eyes with mCNV. This finding suggests that, in eyes with longer axial length, reliance on aqueous concentration alone may underestimate the true secretory burden, whereas vitreous total mass may more reliably reflect the intraocular pathologic state. Significant intergroup differences in the vitreous total mass of other inflammatory mediators, including IL-8 and interferon-γ-induced protein-10 (IP-10), further support immune-inflammatory activation in high myopia and its associated complications (29). Collectively, these findings support the presence of a sustained immune-inflammatory response in myopia.

Compared with intraocular fluid studies, direct histologic and immunopathologic evidence from human tissues remains limited. Jonas et al. performed direct histologic measurements in human globes and compared ocular tissues from patients with primary high myopia (PHM) and secondary high axial myopia (SHM). They found significant thinning of the choroid and posterior sclera in both groups, with these changes being closely associated with axial elongation. In the SHM group, Bruch’s membrane was thinner, suggesting that different etiologies may lead to microstructural differences in posterior segment tissues (30). Although this study did not include immune cell marker analysis, it provides an important reference for future human tissue–based studies investigating the relationship between choroidal degeneration and inflammation in conjunction with immune cell phenotyping.

Based on the evidence summarized above, this review focuses on clinical and experimental studies relevant to the coexistence of choroidal thinning and macrophage-associated immune activation in high myopia. We discuss two major aspects: first, the upstream mechanisms that may trigger macrophage recruitment and state transition, including biomechanical stress and hypoxic signaling; and second, how macrophages after state transition or functional dysregulation may participate in downstream choroidal remodeling. By integrating mechanical stress, metabolic alterations, and immune-inflammatory mechanisms into a single framework, this review aims to cautiously evaluate the rationale of current research models, highlight unresolved questions, and discuss the potential therapeutic implications of targeting macrophage states.

## 2 Upstream mechanisms: signal transduction from biomechanics to polarization signals

### 2.1 Initiating factors: the microenvironment specific to high myopia

A core pathologic feature of high myopia is excessive axial elongation, which places the posterior pole (particularly the macular region) under chronic abnormal biomechanical stress, including stretch, compression, and local redistribution of tissue stress. At the same time, axial elongation may exceed the compensatory capacity of the choroidal vascular system, resulting in a relative reduction in choroidal perfusion pressure and chronic tissue hypoxia. Together, this combination of mechanical overload and chronic hypoxia/metabolic stress constitutes the fundamental microenvironment of the posterior pole in high myopia and may serve as an upstream driver of immune activation and tissue remodeling.

### 2.2 Cellular sensing of signals

The retinal pigment epithelium (RPE), located above Bruch’s membrane and adjacent to the choriocapillaris, is a key structural component of the outer blood-retinal barrier and may function as an amplifier of mechanical and metabolic stimuli. RPE cells express multiple mechanosensitive molecules, including integrins (e.g., αvβ5) and downstream focal adhesion kinase (FAK)-associated signaling complexes, as well as mechanosensitive ion channels such as Piezo1/2, which can sense basement membrane stretch or stiffness changes and convert them into intracellular signals (31, 32). Mechanical stimulation can rapidly activate inflammatory pathways, including NF-κB and JNK, induce RPE secretion of chemotactic mediators such as IL-8 and MCP-1 (CCL2), and upregulate matrix-regulatory molecules such as TIMP-1, thereby promoting local immune cell recruitment and altering matrix metabolism (33–35). In addition, mechanical stimulation may enhance the expression of proangiogenic genes through the SRC-p38-HIF-1α axis, thereby coupling mechanical input to hypoxia-responsive signaling (36).

Choroidal stromal cells and fibroblasts may act as key transducers of mechanical stretch signals. Choroidal stromal cells, particularly fibroblast-like cells, are essential for maintenance of the extracellular matrix (ECM) and vascular support and are highly sensitive to abnormal biomechanical stress. *In vitro* studies have shown that cyclic stretch increases MMP-2 secretion and suppresses TIMP-2 expression in fibroblasts, thereby promoting collagen degradation and matrix relaxation (37). The integrin-actin signaling axis appears to be central to this process and may simultaneously drive cytoskeletal remodeling and upregulation of adhesion-related molecular networks, facilitating recruitment of CCR2+ monocytes. In addition, a microenvironment enriched in matrix degradation products may further influence macrophage polarization bias, thereby creating conditions that favor subsequent inflammation-remodeling coupling.

Macrophages themselves are also capable of sensing both mechanical and hypoxic cues and may function as direct responders within this microenvironment. The Piezo1 ion channel can convert changes in matrix stiffness into Ca2+-dependent signaling that regulates proinflammatory and anti-inflammatory responses (38). Hypoxia-inducible factor-1α (HIF-1α) is a central regulator of macrophage responses to chronic hypoxia and can drive glycolysis and reactive oxygen species (ROS) generation, thereby promoting transcription of proinflammatory mediators such as TNF-α and IL-1β (39, 40). In the stretch-hypoxia microenvironment of high myopia, macrophages may receive concurrent mechanical and hypoxic signals, increasing their tendency to adopt proinflammatory/pro-remodeling phenotypes. This provides a plausible immunobiological explanation for choriocapillaris degeneration and stromal collapse.

Thus, in high myopia, chronic mechanical stretch and relative hypoperfusion at the posterior pole may be jointly sensed by RPE cells, choroidal vascular endothelial cells, stromal cells, and immune cells, leading to convergence of biomechanical and hypoxic-metabolic stress signals across multiple pathways.

### 2.3 Release of polarization cues

The CCL2 (MCP-1)–CCR2 axis is a classical pathway for inflammatory monocyte recruitment and can drive circulating monocytes to migrate into tissues and replenish the macrophage pool (41). In myopia animal models, Zhao et al. observed increased scleral macrophage density and elevated MMP-2 expression in form-deprivation myopic mice, and scleral CCL2 was elevated during myopia progression. Fibroblast-specific deletion of CCL2 suppressed the increase in macrophage density and attenuated myopia development (42), suggesting that the pathway “stromal cells → CCL2 upregulation → monocyte recruitment → macrophage participation in remodeling” is feasible.

After monocytes/macrophages enter local tissues, damage-associated molecular patterns (DAMPs) released during mechanical injury and cellular stress (e.g., HMGB1 and ATP) may amplify TLR/NF-κB and related pathways through pattern recognition receptors (PRRs). In parallel, hypoxia may stabilize proinflammatory transcriptional programs through the glycolysis-HIF-1α axis, thereby biasing macrophages toward proinflammatory/pro-remodeling phenotypes (43–45). By contrast, signals that promote reparative M2-like responses, including IL-4, IL-13, IL-10, and TGF-β, may be insufficient, counterbalanced, or functionally shifted in the chronic stretch-hypoxia microenvironment. This may lead to an imbalance between proinflammatory signaling and repair/remodeling programs, ultimately coupling persistent mechanical abnormalities to a positive feedback loop of inflammatory amplification and structural degeneration.

### 2.4 Autonomic inputs and retinal neuromodulators as upstream modulators of choroidal remodeling

The choroid is not only a vascular tissue but also a densely innervated neurovascular interface. Accumulating evidence indicates that autonomic inputs and retinal neuromodulators can dynamically regulate choroidal thickness and blood flow, thereby influencing the local microenvironment relevant to myopia development. Nitric oxide has long been implicated in visually driven choroidal thickening, dopaminergic signaling is closely linked to ocular growth inhibition and relative choroidal thickening, and atropine-responsive pathways are frequently associated with increases in choroidal thickness in both experimental and clinical settings (7). In humans, parasympatholytic stimulation with homatropine has been shown to increase subfoveal and parafoveal choroidal thickness, further supporting a role for autonomic regulation in shaping the choroidal state (46). Taken together, these observations suggest that neural signals should be considered, alongside mechanical stress and hypoxia, as upstream modulators of the choroidal microenvironment.

These signals may be relevant to macrophage biology through at least two non-mutually exclusive mechanisms. First, they may indirectly influence macrophage programming by altering choroidal perfusion, vascular permeability, and tissue oxygenation. Second, evidence from broader neuroimmune research suggests that autonomic neurotransmitters can directly modulate macrophage phenotype, with β-adrenergic signaling generally associated with reduced pro-inflammatory output and α7 nicotinic acetylcholine receptor signaling linked to anti-inflammatory macrophage responses (47, 48). Although such mechanisms have not yet been demonstrated specifically in the myopic human choroid, they provide a biologically plausible link between retinal neuromodulators, dynamic regulation of choroidal thickness, and immune remodeling. Within this framework, reduced choroidal perfusion or altered autonomic balance may favor a more inflammatory macrophage state, whereas signals associated with choroidal thickening and improved perfusion may support homeostatic or pro-reparative programs.

## 3 Downstream mechanisms: effector functions of polarized macrophages in choroidal remodeling

### 3.1 M1 macrophages: key effectors of destructive remodeling

Within the chronic stretch-hypoxia microenvironment of high myopia, if macrophages shift toward a proinflammatory M1 state, they may serve as contributors to cells for choroidal thinning rather than as proven sole drivers. M1 macrophages release mediators such as TNF-α and IL-1β; in related endothelial systems, these cytokines can damage vascular endothelial cells and disrupt vascular integrity. Cautiously extrapolated to the choroid, such signaling may contribute to reduced vascular density and lower CVI (42, 49). In addition, macrophage-associated matrix metalloproteinases (MMP-2, MMP-9, and MMP-12) may promote degradation of collagen and elastic fiber networks within the choroidal stroma, thereby weakening extracellular matrix support and predisposing the stroma to collapse (50).

Evidence from human aqueous humor studies further supports this concept. With increasing axial length or myopia severity, markers related to inflammation/oxidative stress and proteolysis are elevated and correlate with axial length, suggesting a trend toward concomitant enhancement of inflammatory and proteolytic activity in the intraocular microenvironment of high myopia (51).

### 3.2 M2 macrophages: dual risks of impaired repair and maladaptive repair

Unlike M1 macrophages, M2 responses in pathological settings may pose two related problems. First, reparative activity itself may be insufficient to counter persistent inflammatory injury. Second, the reparative program may become maladaptive and contribute to fibrosis or aberrant angiogenesis. Animal studies have shown that CSF1R blockade markedly reduces choroidal macrophages and leads to progressive choroidal thinning, vascular atrophy, and RPE disorganization; after macrophage repopulation, these abnormalities can be partially reversed. This suggests that resident choroidal macrophages normally provide trophic and homeostatic support to the RPE-choroid unit. Therefore, reduction in macrophage number or impairment of macrophage function may itself contribute to structural degeneration and thinning, indicating that not all macrophage activity is deleterious. On the other hand, within a persistently proinflammatory microenvironment, the anti-inflammatory and reparative functions of M2 macrophages may be suppressed, redirected, or become excessive. Applied cautiously to PM/MMD, this framework may help explain why some “repair-like” responses fail to restore normal choroidal architecture and, at certain stages, may instead accompany fibrosis, atrophy, or a permissive environment for myopic choroidal neovascularization (52, 53).

### 3.3 Cross-cellular communication networks: a positive feedback loop of recruitment, activation, injury, and re-recruitment

The pathogenic effects of macrophages are unlikely to occur in isolation and are more plausibly mediated through cross-cellular positive feedback networks. Under stress conditions, RPE cells, vascular endothelial cells, and stromal cells upregulate recruitment-adhesion signals, including CCL2 (MCP-1) and ICAM-1/VCAM-1, thereby promoting monocyte infiltration and replenishment of the local macrophage pool. Studies of intraocular fluids and peripheral blood have shown that levels of MCP-1, soluble ICAM-1 (sICAM-1), and related molecules are significantly associated with lesion severity and axial length (28, 54).

**FIGURE 2:**
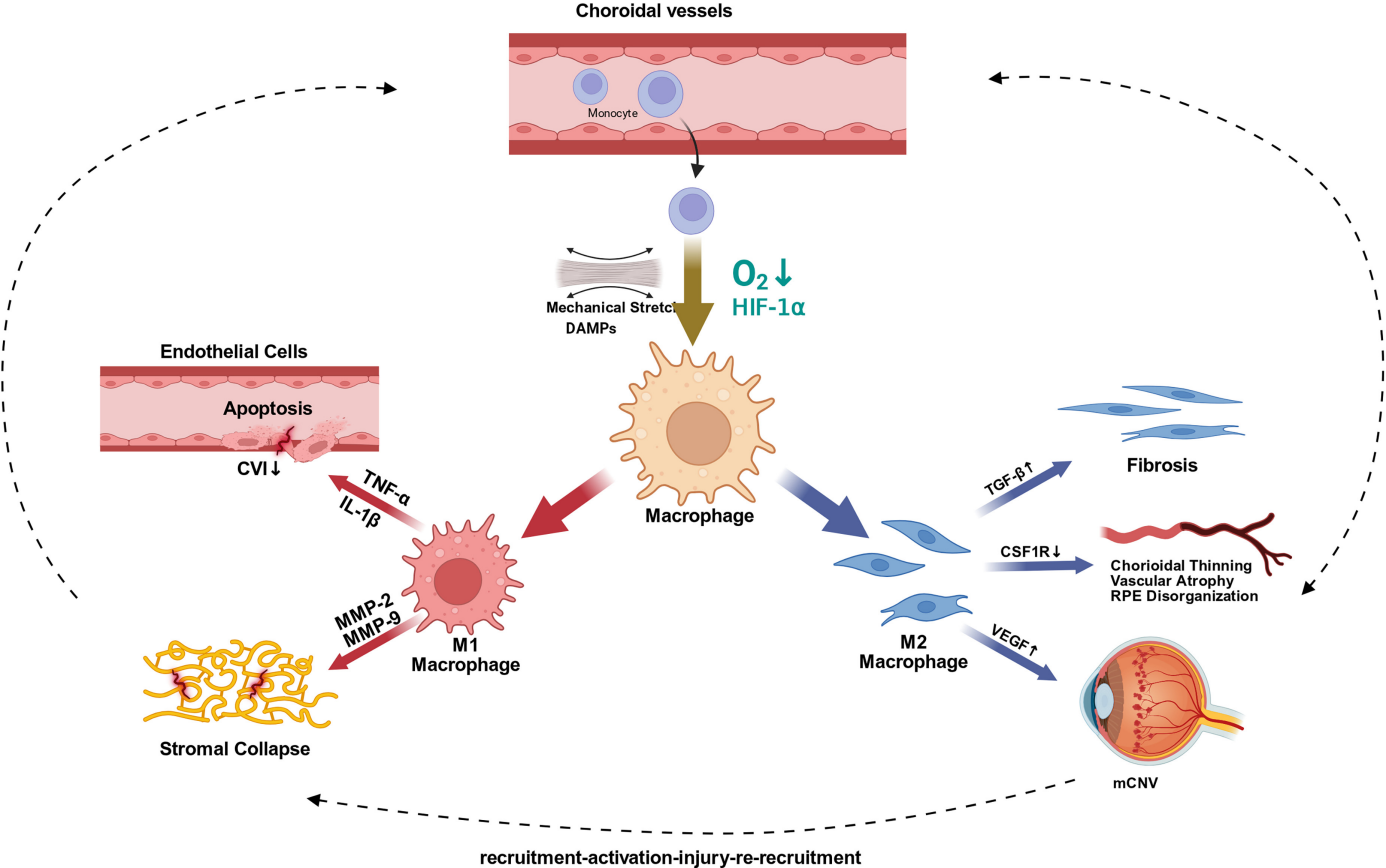
Proposed macrophage-centered immuno-inflammatory remodeling model in high myopia. Under the influence of hypoxia-inducible factor-1α (HIF-1α) and damage-associated molecular patterns (DAMPs), macrophages polarize toward M1 and M2 phenotypes. M1 macrophages may contribute to destructive remodeling through release of proinflammatory cytokines and matrix metalloproteinases (MMPs), are associated with choroidal capillary endothelial injury, reduced choroidal vascularity index (CVI), and stromal matrix collapse. M2 macrophages contribute to maladaptive repair, characterized by impaired trophic support, TGF-β-driven fibrosis, and VEGF-associated myopic choroidal neovascularization (mCNV). Injured tissue further amplifies local adhesion and chemotactic signaling, forming a positive feedback loop of recruitment-activation-injury-re-recruitment. Created with BioRender.com.

After entering the tissue, proinflammatory macrophages can disrupt endothelial integrity through TNF-α, IL-1β, and related mediators, while further amplifying inflammatory signaling through positive feedback. At the same time, these cells degrade extracellular matrix through the MMP-2/MMP-9 axis. In parallel, deficiency or functional deviation of resident/reparative macrophages (including TGF-β- and VEGF-related profibrotic and aberrant angiogenic responses) may weaken maintenance of RPE-choroid homeostasis. Together, these processes may form a positive feedback loop of recruitment-activation-injury-re-recruitment, consistent with progressive choroidal vascular decline and choroidal thinning; however, the current evidence primarily supports an association and biological plausibility rather than an established unidirectional causal chain (29, 42, 52) Figure 2.

Recent studies further suggest that myopia may be accompanied by systemic immune alterations. Meng et al. used single-cell sequencing to construct a multiorgan transcriptomic atlas in myopic mice, spanning ocular tissues (including the retina and choroid) and extraocular organs (including the brain, blood, bone marrow, spleen, thymus, gut, liver, kidney, lung, and adrenal gland). They found widespread immune alterations in myopic mice, including significant macrophage expansion across multiple tissues and enhanced macrophage-mediated intercellular communication. Macrophages from different tissues shared a cross-tissue proinflammatory phenotype driven by hypoxia signaling pathways (HIF-1α), and this feature was also validated in blood samples from patients with myopia (55). This study provides the first whole-organism-level evidence of immune alterations associated with myopia, is consistent with widespread macrophage expansion and a proinflammatory macrophage signature across ocular and extraocular tissues, including findings relevant to the choroid, retina, and multiple extraocular organs, and extends the pathophysiologic framework of myopia from local tissue remodeling toward systemic immunometabolic dysregulation.

### 3.4 Microglia versus choroidal macrophages and additional immune pathways in myopic remodeling

Although both microglia and choroidal macrophages belong to the mononuclear phagocyte system, they should not be regarded as interchangeable in myopia. Retinal microglia reside within the neural retina behind the blood–retinal barrier, whereas the choroid is a highly vascularized connective tissue compartment enriched not only in macrophages but also in dendritic cells and mast cells (56). These distinct tissue environments are likely to support different surveillance and effector programs (56). In high myopia, retinal single-cell transcriptomic studies suggest enhanced inflammatory responsiveness in microglia, whereas direct evidence for human myopic choroidal macrophages remains much more limited (26).

Complement signaling may represent another underappreciated inflammatory pathway in myopic remodeling and its complications. In highly myopic and pathologically myopic eyes, aqueous humor studies have identified increased levels of complement-related proteins, including CFH and multiple classical and alternative pathway components (57). Complement activation has also been linked to retinal neuronal and vascular degeneration, suggesting that it may contribute not only to inflammatory amplification but also to tissue injury and remodeling (58). However, most currently available data come from aqueous humor profiling or retinal readouts rather than direct choroidal mechanistic studies, and its role in shaping choroidal macrophage states remains to be clarified.

A macrophage-centered framework may also overlook other immune cell populations positioned to influence choroidal remodeling. The choroid normally contains abundant mast cells, many of which are located near larger vessels and the choriocapillaris, consistent with potential roles in vascular regulation, permeability, and extracellular matrix remodeling (56). Recent experimental evidence suggests that choroidal mast-cell degranulation can promote choroidal thinning and myopia progression, whereas mast-cell depletion or stabilization suppresses these changes (59). By contrast, direct evidence for adaptive lymphocytes in myopic choroidal remodeling remains limited, although links between allergic inflammation and myopia progression raise the possibility that lymphocyte-associated immune programs may modulate tissue remodeling or related complications such as neovascularization and fibrosis (60).

Taken together, these observations suggest that myopic remodeling is unlikely to be explained by macrophages alone. Rather, it may involve coordinated interactions among retinal microglia, choroidal macrophages, complement pathways, mast cells, and possibly adaptive lymphocyte-associated immune programs. At present, however, the evidence supporting these additional pathways remains uneven, and most should still be interpreted as hypothesis-generating rather than definitive choroid-specific mechanisms.

## 4 Research gaps and future directions: toward precision immunomodulation

### 4.1 Current limitations in knowledge

#### 4.1.1 The evidence chain from “correlation” to “necessity” needs improvement

Current studies provide theoretical support for the mechanical stress–immune-inflammatory–remodeling framework, but the available evidence largely supports an association between macrophage polarization and choroidal thinning in high myopia rather than a definitive causal requirement. *In vivo* studies using choroid-specific, macrophage subset–specific manipulation (rather than global macrophage depletion) remain limited. At present, the strongest *in vivo* evidence is mainly based on changes in overall macrophage presence/absence or total macrophage number (52). In addition, traditional marker systems summarized by the M1/M2 dichotomy may obscure functionally important macrophage subsets, and it remains unclear which subset(s) play dominant pathogenic or protective roles. Myopia-related single-cell studies suggest that macrophages represent a multistate lineage coupled to hypoxia and metabolic signaling (55). Therefore, future studies should perform more refined macrophage subclustering and spatial localization analyses at the choroidal level.

#### 4.1.2 Limitations of choroidal data in human pathology

Most current mechanistic inferences are derived from animal models, whereas important differences exist between humans and animals (e.g., mice) in choroidal structure, vascular architecture, and immune system organization. Human RPE-choroid single-cell RNA atlases have already been established and have revealed regional and age-related differences, as well as intercellular communication networks (61, 62). These studies provide an important methodological foundation for high-resolution immune phenotyping in human tissues. However, choroidal tissue studies from patients with high myopia remain scarce, which limits extrapolation of key mechanistic conclusions and hinders clinical translation.

### 4.2 Future research perspectives

In human tissue–based studies, future work could combine single-cell RNA sequencing (scRNA-seq) and spatial transcriptomics to analyze choroidal tissues from highly myopic and control eyes, and correlate these findings with quantitative OCT/OCTA parameters (e.g., choroidal thickness, CVI, and perfusion-related indices) and major complications such as choroidal neovascularization. In parallel, continued advances in image-based vascular quantification, including automated and AI-assisted analytical approaches, may improve the extraction of reproducible vascular biomarkers from fundus and OCT/OCTA imaging. Such developments could strengthen clinicopathologic correlation and facilitate more refined longitudinal monitoring in myopia research (63). Spatial transcriptomics has already demonstrated value in identifying cellular sources of lesions and molecular pathways in RPE/choroid-related diseases (64). This establishes a more direct translational link between the status of immune cells at the tissue level and clinical imaging phenotypes.

In animal models of myopia, future studies may selectively ablate choroidal macrophages or use polarization-specific inhibitors/agonists to modulate macrophage polarization direction and then assess the effects on choroidal thickness, vascular density, and scleral remodeling. Lineage-tracing approaches may also help define the origin and fate of distinct macrophage subsets during disease progression. These findings may further support development of local ocular drug delivery systems (e.g., nanoparticles and hydrogels) (65) to achieve choroidal enrichment and sustained release, thereby improving treatment safety and efficacy. Such immunomodulatory strategies must be approached with caution, as systemic or prolonged local inhibition of pathways such as CSF1R or CCR2 carries the risk of disrupting homeostatic macrophage function, potentially compromising the structural integrity of the RPE-choroid complex or increasing susceptibility to ocular inflammation. Future studies will need to balance efficacy against such safety considerations, ideally through targeted drug delivery systems that achieve choroidal enrichment while minimizing systemic exposure.

At the clinical level, it will also be important to evaluate whether immunomodulatory strategies can be combined with current myopia-control approaches (e.g., low-dose atropine and optical defocus interventions) to enable multitarget regulation of myopia progression and complication risk.

## 5 Discussion

Choroidal thinning in high myopia should not be regarded solely as an atrophic change caused by passive mechanical stretch or reduced perfusion, but rather as an active and dynamic remodeling process. In this review, we propose a mechanistic framework centered on mechanical stretch/relative hypoxic stress–immune microenvironment imbalance–macrophage polarization–destructive choroidal remodeling, integrating evidence from ocular imaging, intraocular fluid analyses, and animal studies. Within this framework, immune-inflammatory mechanisms–particularly macrophage polarization–may play important roles in the development and progression of high myopia and in the formation of related complications. As some of the evidence is still derived from extrapolations from studies of the sclera, retina/RPE or systemic immunity, this framework should currently be understood primarily as an integrative model proposing choroid-specific hypotheses, with the aim of providing new insights into the pathogenesis of high myopia.

Despite important advances, major challenges remain, including an incomplete evidence chain and the limited availability of human tissue–based data. However, with the continued application of emerging technologies such as single-cell omics, spatial biology, and gene editing, future studies may clarify the causal relationship between choroidal immune ecology and structural degeneration, identify clinically translatable regulatory nodes, and provide a foundation for the development of diagnostic biomarkers and targeted interventions centered on macrophage polarization. Such efforts may ultimately offer new strategies for the prevention and treatment of high myopia.

## References

[1] Holden BA Fricke TR Wilson DA Jong M Naidoo KS Sankaridurg P et al. Global prevalence of myopia and high myopia and temporal trends from 2000 through 2050. *Ophthalmology.* (2016) 123:1036–42. 10.1016/j.ophtha.2016.01.006 26875007

[2] Flitcroft DI He M Jonas JB Jong M Naidoo K Ohno-Matsui K et al. IMI—Defining and classifying myopia: a proposed set of standards for clinical and epidemiologic studies. *Invest Ophthalmol Vis Sci.* (2019) 60:M20–30. 10.1167/iovs.18-25957 30817826 PMC6735818

[3] Ohno-Matsui K Wu PC Yamashiro K Vutipongsatorn K Fang Y Cheung CMG et al. IMI pathologic myopia. *Invest Ophthalmol Vis Sci.* (2021) 62:5. 10.1167/iovs.62.5.5 33909033 PMC8083114

[4] Ikuno Y Tano Y. Retinal and choroidal biometry in highly myopic eyes with spectral-domain optical coherence tomography. *Invest Ophthalmol Vis Sci.* (2009) 50:3876–80. 10.1167/iovs.08-3325 19279309

[5] Ramrattan RS van der Schaft TL Mooy CM de Bruijn WC Mulder PG de Jong PT. Morphometric analysis of Bruch’s membrane, the choriocapillaris, and the choroid in aging. *Invest Ophthalmol Vis Sci.* (1994) 35:2857–64. 8188481

[6] Wildsoet C Wallman J. Choroidal and scleral mechanisms of compensation for spectacle lenses in chicks. *Vis Res.* (1995) 35:1175–94. 10.1016/0042-6989(94)00233-c 7610579

[7] Ostrin LA Harb E Nickla DL Read SA Alonso-Caneiro D Schroedl F et al. IMI—The dynamic choroid: new insights, challenges, and potential significance for human myopia. *Invest Ophthalmol Vis Sci.* (2023) 64:4. 10.1167/iovs.64.6.4 37126359 PMC10153586

[8] Spaide RF Koizumi H Pozzoni MC. Enhanced depth imaging spectral-domain optical coherence tomography. *Am J Ophthalmol.* (2008) 146:496–500. 10.1016/j.ajo.2008.05.032 18639219

[9] Zhou LX Shao L Xu L Wei WB Wang YX You QS. The relationship between scleral staphyloma and choroidal thinning in highly myopic eyes: the Beijing Eye Study. *Sci Rep.* (2017) 7:9825. 10.1038/s41598-017-10660-z 28852194 PMC5575118

[10] Zhu H Liu C Gao M Zhang S Zhang L Zhao Q. Choroidal thickness in relation to diopter and axial length among myopic children. *Front Med.* (2023) 10:1241352. 10.3389/fmed.2023.1241352 37928462 PMC10623004

[11] Zhou ZH Xiong PP Sun J Wang YL Wang JL. Effects of posterior staphyloma on choroidal structure in myopic adults: a retrospective study. *BMC Ophthalmol.* (2023) 23:406. 10.1186/s12886-023-03158-y 37814232 PMC10563244

[12] Shao L Zhao H Zhang R Zhou W Wei WB. Distribution and associated factors of choroidal thickness in highly myopic eyes—A real-world study based on a Chinese population. *Eye.* (2025) 39:102–8. 10.1038/s41433-024-03383-9 39448852 PMC11733017

[13] Nishida Y Fujiwara T Imamura Y Lima LH Kurosaka D Spaide RF. Choroidal thickness and visual acuity in highly myopic eyes. *Retina.* (2012) 32:1229–36. 10.1097/IAE.0b013e318242b990 22466466

[14] Harb E Hyman L Gwiazda J Marsh-Tootle W Zhang Q Hou W et al. Choroidal thickness profiles in myopic eyes of young adults in the correction of myopia evaluation trial cohort. *Am J Ophthalmol.* (2015) 160:62–71.e2. 10.1016/j.ajo.2015.04.018 25896460 PMC4465039

[15] Song Y Tham YC Chong C Ong R Fenner BJ Cheong KX et al. Patterns and determinants of choroidal thickness in a multiethnic Asian population: the Singapore Epidemiology of Eye Diseases Study. *Ophthalmol Retina.* (2021) 5:458–67. 10.1016/j.oret.2020.08.012 32858246

[16] Tan CS Cheong KX Lim LW Li KZ. Topographic variation of choroidal and retinal thicknesses at the macula in healthy adults. *Br J Ophthalmol.* (2014) 98:339–44. 10.1136/bjophthalmol-2013-304000 24288389

[17] Li Z Long W Hu Y Zhao W Zhang W Yang X. Features of the choroidal structures in myopic children based on image binarization of optical coherence tomography. *Invest Ophthalmol Vis Sci.* (2020) 61:18. 10.1167/iovs.61.4.18 32298436 PMC7401716

[18] Ding X Wang Y Wan N Wu X Shen J Wang H et al. Elevated nasal-temporal asymmetry of the choroidal vascular index in pathologically myopic eyes. *Invest Ophthalmol Vis Sci.* (2025) 66:66. 10.1167/iovs.66.12.66 41020555 PMC12489860

[19] Xu Y Yang W Long T Shang W Xu X Wang J et al. Analysis of microcirculation changes in the macular area and para-optic disk region after implantable collamer lens implantation in patients with high myopia. *Front Neurosci.* (2022) 16:867463. 10.3389/fnins.2022.867463 35663554 PMC9160968

[20] Liu Y Wang L Xu Y Pang Z Mu G. The influence of the choroid on the onset and development of myopia: from perspectives of choroidal thickness and blood flow. *Acta Ophthalmol.* (2021) 99:730–8. 10.1111/aos.14773 33550704

[21] Xu R Zheng J Liu L Zhang W. Effects of inflammation on myopia: evidence and potential mechanisms. *Front Immunol.* (2023) 14:1260592. 10.3389/fimmu.2023.1260592 37849748 PMC10577208

[22] Zhang J Kamoi K Zong Y Yang M Zou Y Ohno-Matsui K. Inflammation and immune pathways in myopia: an overview on pathomechanisms and treatment prospects. *Clin Rev Allergy Immunol.* (2025) 68:98. 10.1007/s12016-025-09094-7 41191163 PMC12589388

[23] Murray PJ Wynn TA. Protective and pathogenic functions of macrophage subsets. *Nat Rev Immunol.* (2011) 11:723–37. 10.1038/nri3073 21997792 PMC3422549

[24] Murray PJ Allen JE Biswas SK Fisher EA Gilroy DW Goerdt S et al. Macrophage activation and polarization: nomenclature and experimental guidelines. *Immunity.* (2014) 41:14–20. 10.1016/j.immuni.2014.06.008 25035950 PMC4123412

[25] Zheng B Cui D Deng B Long W Ye G Zhang S et al. Form-deprivation myopia promotes sclera M2-type macrophage polarization in mice. *Biochem Biophys Res Commun.* (2024) 737:150490. 10.1016/j.bbrc.2024.150490 39146710

[26] Yao Y Chen Z Wu Q Lu Y Zhou X Zhu X. Single-cell RNA sequencing of retina revealed novel transcriptional landscape in high myopia and underlying cell-type-specific mechanisms. *MedComm.* (2023) 4:e372. 10.1002/mco2.372 37746666 PMC10511833

[27] Mérida S García-Gen E Desco C Navea A Bosch-Morell F. Hypoxia and growth factor profiling in high myopia: linking HIF-1α suppression and PDGF-BB activation to structural degeneration. *Front Med.* (2025) 12:1705777. 10.3389/fmed.2025.1705777 41438149 PMC12719478

[28] Han X Hu Y Chen Y Cai J Chen Y Li N et al. Expression of cytokines in the aqueous humor of cataract patients with pathologic myopia and simple high myopia. *Mol Vis.* (2024) 30:369–77. 39959181 PMC11829778

[29] Zhang S Mao J Chen N Fang Y Chen Y Zheng Z et al. Difference in aqueous concentration and vitreous mass of cytokines in high myopias with and without choroidal neovascularization. *Front Med.* (2022) 9:1029425. 10.3389/fmed.2022.1029425 36438065 PMC9684635

[30] Jonas JB Holbach L Panda-Jonas S. Histologic differences between primary high myopia and secondary high myopia due to congenital glaucoma. *Acta Ophthalmol.* (2016) 94:147–53. 10.1111/aos.12937 26695106

[31] Nandrot EF Kim Y Brodie SE Huang X Sheppard D Finnemann SC. Loss of synchronized retinal phagocytosis and age-related blindness in mice lacking αvβ5 integrin. *J Exp Med.* (2004) 200:1539–45. 10.1084/jem.20041447 15596525 PMC2211990

[32] Finnemann SC. Focal adhesion kinase signaling promotes phagocytosis of integrin-bound photoreceptors. *EMBO J.* (2003) 22:4143–54. 10.1093/emboj/cdg416 12912913 PMC175805

[33] Yoshida A Elner SG Bian ZM Elner VM. Induction of interleukin-8 in human retinal pigment epithelial cells after denuding injury. *Br J Ophthalmol.* (2001) 85:872–6. 10.1136/bjo.85.7.872 11423465 PMC1724031

[34] Gao M Wu S Ji J Zhang J Liu Q Yue Y et al. The influence of actin depolymerization induced by cytochalasin D and mechanical stretch on interleukin-8 expression and JNK phosphorylation levels in human retinal pigment epithelial cells. *BMC Ophthalmol.* (2017) 17:43. 10.1186/s12886-017-0437-z 28388885 PMC5384187

[35] Yamaguchi K Matsuo T Shiraga F Ohtsuki H. TIMP-1 production by bovine retinal pigment epithelial cells increases in response to cyclic mechanical stretch. *Jpn J Ophthalmol.* (2001) 45:470–4. 10.1016/s0021-5155(01)00379-3 11583667

[36] Ashimori A Higashijima F Ogata T Sakuma A Hamada W Sunada J et al. HIF-1α-dependent upregulation of angiogenic factors by mechanical stimulation in retinal pigment epithelial cells. *Dis Model Mech.* (2024) 17:dmm050640. 10.1242/dmm.050640 38691000 PMC11095633

[37] Shelton L Rada JS. Effects of cyclic mechanical stretch on extracellular matrix synthesis by human scleral fibroblasts. *Exp Eye Res.* (2007) 84:314–22. 10.1016/j.exer.2006.10.004 17123515 PMC2583333

[38] Atcha H Jairaman A Holt JR Meli VS Nagalla RR Veerasubramanian PK et al. Mechanically activated ion channel Piezo1 modulates macrophage polarization and stiffness sensing. *Nat Commun.* (2021) 12:3256. 10.1038/s41467-021-23482-5 34059671 PMC8167181

[39] O’Neill LAJ Pearce EJ. Immunometabolism governs dendritic cell and macrophage function. *J Exp Med.* (2016) 213:15–23. 10.1084/jem.20151570 26694970 PMC4710204

[40] Shao C Lin S Liu S Jin P Lu W Li N et al. HIF1α-induced glycolysis in macrophage is essential for the protective effect of ouabain during endotoxemia. *Oxid Med Cell Longev.* (2019) 2019:7136585. 10.1155/2019/7136585 31182997 PMC6512009

[41] Shi C Pamer EG. Monocyte recruitment during infection and inflammation. *Nat Rev Immunol.* (2011) 11:762–74. 10.1038/nri3070 21984070 PMC3947780

[42] Zhao F Wu H Reinach PS Wu Y Zhai Y Lei Y et al. Up-regulation of matrix metalloproteinase-2 by scleral monocyte-derived macrophages contributes to myopia development. *Am J Pathol.* (2020) 190:1888–908. 10.1016/j.ajpath.2020.06.002 32553806

[43] Chen R Kang R Tang D. The mechanism of HMGB1 secretion and release. *Exp Mol Med.* (2022) 54:91–102. 10.1038/s12276-022-00736-w 35217834 PMC8894452

[44] Dang B Gao Q Zhang L Zhang J Cai H Zhu Y et al. The glycolysis/HIF-1α axis defines the inflammatory role of IL-4-primed macrophages. *Cell Rep.* (2023) 42:112471. 10.1016/j.celrep.2023.112471 37149865

[45] Chen S Saeed AFUH Liu Q Jiang Q Xu H Xiao GG et al. Macrophages in immunoregulation and therapeutics. *Signal Transduct Target Ther.* (2023) 8:207. 10.1038/s41392-023-01452-1 37211559 PMC10200802

[46] Sander BP Collins MJ Read SA. The effect of topical adrenergic and anticholinergic agents on the choroidal thickness of young healthy adults. *Exp Eye Res.* (2014) 128:181–9. 10.1016/j.exer.2014.10.003 25304219

[47] Freire BM Melo FM Basso AS. Adrenergic signaling regulation of macrophage function: do we understand it yet? *Immunother Adv.* (2022) 2:ltac010. 10.1093/immadv/ltac010 36284839 PMC9585663

[48] Keever KR Yakubenko VP Hoover DB. Neuroimmune nexus in the pathophysiology and therapy of inflammatory disorders: role of α7 nicotinic acetylcholine receptors. *Pharmacol Res.* (2023) 191:106758. 10.1016/j.phrs.2023.106758 37028776 PMC13134767

[49] Barros Ferreira L Ashander LM Ma Y Appukuttan B Williams KA Best G et al. Effects of tumor necrosis factor-α and interleukin-1β on human retinal endothelial cells. *Cytokine.* (2024) 173:156407. 10.1016/j.cyto.2023.156407 37924741

[50] Wu H Chen W Zhao F Zhou Q Reinach PS Deng L et al. Scleral hypoxia is a target for myopia control. *Proc Natl Acad Sci U S A.* (2018) 115:E7091–100. 10.1073/pnas.1721443115 29987045 PMC6064999

[51] Yu Q Wang C Liu Z Yue Y Hsiao Y Zhou Q et al. Association between inflammatory cytokines and oxidative stress levels in aqueous humor with axial length in human myopia. *Exp Eye Res.* (2023) 237:109670. 10.1016/j.exer.2023.109670 37806610

[52] Yang X Zhao L Campos MM Abu-Asab M Ortolan D Hotaling N et al. CSF1R blockade induces macrophage ablation and results in mouse choroidal vascular atrophy and RPE disorganization. *eLife.* (2020) 9:e55564. 10.7554/eLife.55564 32234210 PMC7156269

[53] Zhao Z Zhang Y Zhang C Zhang J Luo X Qiu Q et al. TGF-β promotes pericyte-myofibroblast transition in subretinal fibrosis through the Smad2/3 and Akt/mTOR pathways. *Exp Mol Med.* (2022) 54:673–84. 10.1038/s12276-022-00778-0 35624154 PMC9166792

[54] Zhu X Meng J Han C Wu Q Du Y Qi J et al. CCL2-mediated inflammatory pathogenesis underlies high myopia-related anxiety. *Cell Discov.* (2023) 9:94. 10.1038/s41421-023-00588-2 37699875 PMC10497683

[55] Meng J Zhang Y Zhu M Du Y Yao Y Liu S et al. Single-cell profiling reveals a shared proinflammatory macrophage signature across multiple organs in myopia. *Cell Discov.* (2025) 11:97. 10.1038/s41421-025-00835-8 41330940 PMC12672714

[56] McMenamin PG Saban DR Dando SJ. Immune cells in the retina and choroid: two different tissue environments that require different defenses and surveillance. *Prog Retin Eye Res.* (2019) 70:85–98. 10.1016/j.preteyeres.2018.12.002 30552975 PMC7321801

[57] García-Gen E Penadés M Mérida S Desco C Araujo-Miranda R Navea A et al. High myopia and the complement system: factor H in myopic maculopathy. *J Clin Med.* (2021) 10:2600. 10.3390/jcm10122600 34204630 PMC8231207

[58] Zeng L Li X Pan W Tang Y Lin D Wang M et al. Intraocular complement activation is related to retinal vascular and neuronal degeneration in myopic retinopathy. *Front Cell Neurosci.* (2023) 17:1187400. 10.3389/fncel.2023.1187400 37448698 PMC10336352

[59] Shi J Ikeda SI Fukuchi T Chen J Gettinger K Imanishi S et al. Choroidal mast cells and their degranulation are a pivotal trigger for myopia development. *Invest Ophthalmol Vis Sci.* (2025) 66:22. 10.1167/iovs.66.14.22 41222199 PMC12614256

[60] Wei CC Kung YJ Chen CS Chang CY Lin CJ Tien PT et al. Allergic conjunctivitis-induced retinal inflammation promotes myopia progression. *EBioMedicine.* (2018) 28:274–86. 10.1016/j.ebiom.2018.01.024 29398596 PMC5835569

[61] Voigt AP Mulfaul K Mullin NK Flamme-Wiese MJ Giacalone JC Stone EM et al. Single-cell transcriptomics of the human retinal pigment epithelium and choroid in health and macular degeneration. *Proc Natl Acad Sci U S A.* (2019) 116:24100–7. 10.1073/pnas.1914143116 31712411 PMC6883845

[62] Huang L Ye L Li R Zhang S Qu C Li S et al. Dynamic human retinal pigment epithelium (RPE) and choroid architecture based on single-cell transcriptomic landscape analysis. *Genes Dis.* (2023) 10:2540–56. 10.1016/j.gendis.2022.11.007 37554187 PMC10404887

[63] Chen N Zhu Z Yang W Wang Q. Progress in clinical research and applications of retinal vessel quantification technology based on fundus imaging. *Front Bioeng Biotechnol.* (2024) 12:1329263. 10.3389/fbioe.2024.1329263 38456011 PMC10917897

[64] Voigt AP Mullin NK Navratil EM Flamme-Wiese MJ Lin LC Scheetz TE et al. Gene expression within a human choroidal neovascular membrane using spatial transcriptomics. *Invest Ophthalmol Vis Sci.* (2023) 64:40. 10.1167/iovs.64.13.40 37878301 PMC10615143

[65] Wu Y Li X Fu X Huang X Zhang S Zhao N et al. Innovative nanotechnology in drug delivery systems for advanced treatment of posterior segment ocular diseases. *Adv Sci.* (2024) 11:2403399. 10.1002/advs.202403399 39031809 PMC11348104

